# How important is hidden phenotypic plasticity arising from alternative but converging developmental trajectories, and what limits it?

**DOI:** 10.1242/jeb.246010

**Published:** 2024-03-07

**Authors:** Neil B. Metcalfe

**Affiliations:** School of Biodiversity, One Health & Veterinary Medicine, Graham Kerr Building, University of Glasgow, Glasgow G12 8QQ, UK

**Keywords:** Compensatory growth, Developmental plasticity, Growth, Life history, Resource allocation, Trade-off

## Abstract

Developmental plasticity ­– the capacity for a genotype to develop into different phenotypes, depending on the environment – is typically viewed from the perspective of the resulting phenotype. Thus, if development is viewed as a trajectory towards a target, then developmental plasticity allows environmentally induced alterations to the target. However, there can also be variations in the trajectory. This is seen with compensatory responses, for instance where growth accelerates after an earlier period of food shortage, or where investment in sexual ornaments is maintained even when resources are limiting. If the compensation is complete, the adult phenotype can appear ‘normal’ (i.e. the different developmental trajectories converge on the same target). However, alternative trajectories to a common target can have multiple long-term consequences, including altered physiological programming and rates of senescence, possibly owing to trade-offs between allocating resources to the prioritized trait versus to body maintenance. This suggests that plasticity in developmental trajectories towards a common target leads to variation in the resilience and robustness of the adult body. This form of developmental plasticity is far more hidden than plasticity in final adult target, but it may be more common. Here, I discuss the causes, consequences and limitations of these different kinds of plasticity, with a special focus on whether they are likely to be adaptive. I emphasize the need to study plasticity in developmental trajectories, and conclude with suggestions for future research to tease apart the different forms of developmental plasticity and the factors that influence their evolution and expression.

## Teaser

**Summary:** A potentially common but hidden form of developmental plasticity is variation in developmental trajectory towards a common final phenotype, which can have major lasting effects on physiology and life history.

## Introduction: Compensatory responses as a form of hidden plasticity

**Fig. 1.**
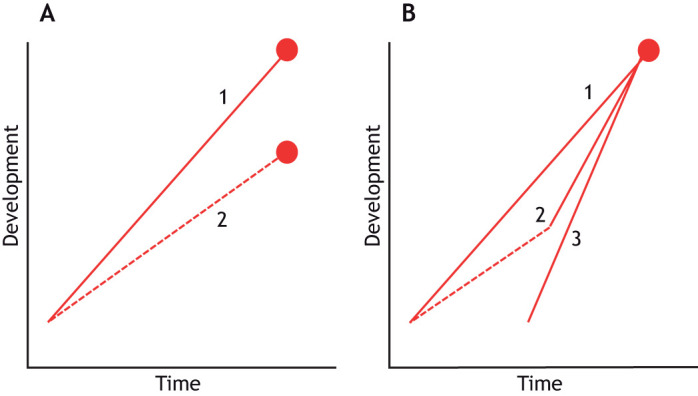
**Developmental trajectories that illustrate developmental plasticity in terms of altered targets and altered trajectories.** A hypothetical developmental trait (such as body size) is plotted on the *y*-axis. (A) Altered targets. In this scenario, two individuals of the same genotype receive contrasting cues of environmental quality (individual 1 sensing a better environment than individual 2); this causes an alteration in the developmental target (indicated by the filled circle). (B) Altered trajectories. In this scenario, the cue indicating a poorer environment is short-lived relative to total development time. A perceived improvement in the environment (indicated by switch in trajectory from dashed to solid line) allows individual 2 to adjust its target partway through development; it then accelerates development and reaches the same target as individual 1. Alternatively, individual 3 develops faster than individuals 1 or 2, possibly due to a perceived time shortage. All three individuals are superficially similar in adulthood but have reached this target by different trajectories. As a result they are ‘built differently’, with potential consequences for later performance.

There are two obvious phenotypic outcomes of parental or early developmental effects. One is the clearly maladaptive damaged phenotype, that arises because of resource limitations (e.g. food shortage leading to stunting) or because normal development has been disrupted (e.g. through genetic mutations arising from exposure to carcinogens or pollutants). The more biologically interesting outcome is where there appears to be a change in the developmental trajectory, leading to a different phenotype which has a higher expected fitness given the current or anticipated environment. Given that development is often framed in the context of leading towards a target (Klingenberg, 2019), this might be termed an adjusted target and is usually presumed to be an adaptive response (Fig. 1A). There are numerous well-studied examples of this form of phenotypic plasticity, known as developmental plasticity (Bateson et al., 2014; Berghänel et al., 2017; Gotthard and Nylin, 1995; MacLeod et al., 2022; Moczek et al., 2011). Phenotypic plasticity is usually defined as different phenotypes being produced in different environments; in the case of developmental plasticity, the environments can be those prevailing before and/or after birth, and can include the environment experienced by parents or even earlier generations (see Box 1 for an attempt to clarify the terminology).

**Box 1. The terminology of developmental plasticity**

The field of developmental plasticity is beset with confusing terminology, not least because it can be hard to determine whether the cue to the response is experienced directly by the developing individual or by its parent(s) (or earlier ancestors). For instance, in mammals, a stressor may simultaneously affect a pregnant female (the parental generation), the female fetus she is carrying (the F1 generation) and the eggs inside that fetus (that will give rise to the F2 generation), so the route by which the stressor affects F2 individuals is far from clear (Burton and Metcalfe, 2014). In order to get around this issue, the most generally used term when more than one generation is potentially involved is transgenerational plasticity (TGP), which refers to any plasticity shown in offspring in response to a cue or environment that is experienced prior to their birth (i.e. by an earlier generation); this is contrasted with within-generation plasticity (WGP) (Donelson et al., 2018). TGP includes situations where the cue was potentially directly experienced by the offspring *in utero*, so is a looser definition than transgenerational epigenetic inheritance (TEI), which presumes that cues were not directly experienced by the offspring but were passed on epigenetically in gametes (Bell and Hellmann, 2019). Although TEI is conceptually a more precise definition than TGP, it is also far less easy to apply to the majority of examples of developmental plasticity, as it can only be proven to exist through long-term controlled experiments lasting three to four generations. Given the difficulty in separating these intergenerational effects and their complex terminology, in this paper I consider transgenerational and early developmental effects together, for the simple reason that in many species it can be hard to determine whether it is the parent or the developing offspring that is responding to an environmental cue.

A further reason why transgenerational effects can be hard to unravel in nature is that they can derive from either parent (Hope et al., 2022; Papatheodoulou et al., 2022), and their impact on offspring can be sex-dependent: for example, the effects of parental stress can depend on the sex of both the parent and the offspring (Hellmann et al., 2020a). These sex×stress×generation interactions become ever more complex by the grandoffspring F2 generation (Hellmann et al., 2020b). This suggests complex transgenerational epigenetic processes that are still poorly understood (Bell and Hellmann, 2019).

However, I suggest that there is a third, more hidden, kind of developmental plasticity. This is the adjusted trajectory, in which the target is unaltered but there is a change in the developmental trajectory by which it is reached. Developmental systems are often characterized by being canalized (i.e. buffered against environmental influences) yet also exhibiting multiple routes by which the same phenotypic outcome can be achieved (i.e. the concept of degeneracy) (Hoke et al., 2019), and there are a range of reasons why the developmental trajectory might differ among individuals. A common one is where development is constrained for a period (usually because of adverse circumstances such as depressed food availability or quality), but if circumstances improve before development is completed, there is then an attempt to mitigate the effect by returning to the original state or trajectory (so that the original and the adjusted developmental trajectories converge on the same target; trajectory B in Fig. 1B). This compensatory response has been most often studied in the context of growth, where a period of slowed growth can be followed by an acceleration or prolongation of growth once conditions improve (Berghänel et al., 2017; Hector and Nakagawa, 2012). Another cause of an adjusted trajectory is a shortage of time. Animals living in seasonal environments may accelerate their rate of development if environmental cues indicate that they are running out of time to complete development (trajectory C in Fig. 1B): thus, larval damselflies have a faster rate of growth if exposed to a late rather than early summer photoperiod (De Block and Stoks, 2004).

These examples show that, although growth and development are sometimes assumed to be at the maximum rate permissible for the prevailing food supply (and temperature in the case of ectotherms), this is not necessarily the case. Both growth and development can be regulated at a submaximal rate, either to match an internal trajectory (for instance that based on expected size or developmental stage for the time of year; Gotthard, 2008) or to suit the local social environment (Buston and Clutton-Brock, 2022), but can be accelerated if conditions change. Growth and developmental rate should therefore be viewed as labile traits that are under selection.

A faster growth rate after a period of adversity or when the available time for development is short suggests that the original target may still have the highest expected fitness. Importantly, this means that if compensation is complete so that the developmental trajectories converge, then the adult phenotype may be superficially indistinguishable from that of ‘normally’ developing conspecifics. The same is true if resource shortages force compromises on the investment in different traits: prioritization of investment into the morphological traits that are most crucial for reproductive success may result in a superficially similar appearance to well-nourished conspecifics, despite the reduced resources. Thus, male fiddler crabs *Uca annulipes* that have to re-grow a lost claw produce one that is of similar size to the original but is lighter in mass; it looks sufficiently like that of other males to be equally attractive to females (Backwell et al., 2000). Male three-spined sticklebacks (*Gasterosteus aculeatus*) that have reduced access to dietary carotenoids in the period before breeding prioritize the allocation of these pigments into their throat tissues rather than the rest of the body, so that they can commence the breeding season looking as colourful, and hence as attractive to potential mates, as well-nourished rivals (Lindström et al., 2009). The plasticity of the adjusted trajectory is thus evident not in the final phenotype but in the route by which this is achieved. Therefore, it may be difficult to determine in later life that this plasticity has occurred, unless there has been documentation of stages in development such as initial size, food intake or growth trajectory (Fig. 1B). Thus, it might be a much more common (but more often undetected) form of developmental plasticity than the usual textbook examples that are based on altered targets. However, it is increasingly clear that these altered trajectories may themselves have significant immediate and long-term consequences, even if they appear to have (nearly) restored the phenotype to the original target (Metcalfe and Monaghan, 2001). In short, the organism that has reached the original target but through an altered trajectory is ‘built differently’, and this can come at a significant cost. This issue of the deferred costs of plasticity will be considered below (see ‘What constrains developmental plasticity?’), but first it is useful to consider the conditions that prompt the development of alternative phenotypes and whether these are actually adaptive.

## The triggers of developmental plasticity

A common cause of intergenerational and early developmental plasticity is stress. For instance, if parents experience environmental stressors just before or during a breeding attempt, this can result in the offspring having a damaged phenotype (Aizer et al., 2016; Eriksen et al., 2015; Sheriff and Love, 2013). The exact nature of the stressor may not be that important, because evidence from both mammals and birds suggests that different kinds of environmental stressors on mothers can have very similar effects on the offspring phenotype; this may be because they trigger a common physiological response through the hypothalamic–pituitary–adrenal axis involving the release of glucocorticoid (‘stress’) hormones such as cortisol or corticosterone (Berghänel et al., 2017; Potticary and Duckworth, 2020).

However, the impact of the stressor can depend on its timing relative to the reproductive attempt. A clear example of this comes from the detailed analyses of the outcome of the Dutch Hunger Winter: this was a short-lived but severe period of wartime famine imposed by the German occupiers on the Dutch civilian population during the winter of 1944–1945. This well-documented and discrete duration of severe food restriction on an otherwise reasonably well-nourished population would undoubtedly have altered developmental trajectories, and has allowed epidemiologists to document the consequences for offspring and grandoffspring of maternal nutritional stress during different periods of gestation. Their analyses have revealed that famine during early gestation was associated with offspring neural tube defects and schizophrenia, famine during late gestation was associated with offspring having a greater risk of type II diabetes in later life, and famine at any time during gestation was associated with offspring having a higher likelihood of adult obesity (Lumey et al., 2011). The mechanism of these effects is not clear, but there is evidence of changes in DNA methylation patterns in gene promotor regions persisting into old age in the offspring, indicating epigenetic modifications and potentially transgenerational epigenetic inheritance (Tobi et al., 2014). The effect of an increased likelihood of obesity in offspring experiencing an adjusted trajectory owing to prenatal famine has been interpreted as the programming of a ‘thrifty phenotype’ that is adapted to a poor nutritional environment (and so stores all excess lipid) (Hales and Barker, 2001). However, it seems unlikely that this would ever have been adaptive, given the tendency for the food supply of ancestral human populations to fluctuate markedly relative to their lifespan, such that the food availability at the time of gestation would be a poor predictor of food supply for the rest of life (Wells, 2012).

One commonly studied environmental stressor is the risk of predation. Parental exposure to predators has been shown to affect offspring phenotypes across a broad range of taxa, affecting a range of traits from morphology through to behaviour (Jonsson et al., 2022; MacLeod et al., 2022). However, there is no general and predictable response, and it is not always clear whether the changes are adaptive (MacLeod et al., 2022). Predators often act as acute stressors, because the period of exposure of the prey to the predator can be brief and so trigger a stress response that is short-lived in duration. In contrast, many other environmental stressors may be chronic in nature – for instance food shortage, or exposure to drought, pollutants or adverse temperatures. Chronic exposure to these stressors may allow adjustment to reduce their impact (e.g. temperature acclimation, a compensatory response). It also allows more time for any phenotypic plasticity to be expressed. A recent example comes from a study of the long-term effect of subjecting female three-spined sticklebacks *Gasterosteus aculeatus* to a diverse range of environmental stressors (so preventing habituation) throughout their long breeding season, over the course of which they produced multiple clutches (Magierecka et al., 2021). There were no significant differences in the phenotype of offspring produced by stressed and control females early in the season. But by the time of the last clutches of the breeding season, the mothers exposed to stressors were producing offspring that were superficially similar to controls but had a higher survival rate (suggesting an altered trajectory), and in turn, these offspring produced larger eggs and fry (the grandoffspring of the exposed mothers) when they themselves came to breed (Magierecka et al., 2022). The physiological mechanisms underlying these effects are not clear, not least because the environmental stressors, although causing changes in the mothers' behaviour, did not cause a measurable increase in their cortisol levels (Magierecka et al., 2021).

An environmental cause of developmental plasticity need not be stressful. There is increasing evidence that temperature during embryo development – which will affect the developmental trajectory – can cause broad-scale changes in the resulting phenotype. This is most evident in ectotherms, owing to the pervasive effect of ambient temperature on their physiological processes (Jonsson and Jonsson, 2019; Jonsson et al., 2022). However, it is also important for birds, where the temperature at which the eggs are incubated is now known to influence post-hatching traits such as metabolic rate (Hope et al., 2022). This may explain why the basal metabolic rate of wintering wild great tits (*Parus major*) is negatively correlated with the ambient minimum temperature they experienced during incubation: it has been suggested that this is adaptive, with the metabolism of embryos experiencing a cold spring being programmed to cope with colder winters (Broggi et al., 2022). This plasticity in metabolic rate may be driven by altered programming of mitochondrial function, because mitochondrial respiration accounts for the majority of whole-animal oxygen consumption; incubation temperature in Japanese quail (*Coturnix japonica*) has been shown to influence mitochondrial function right through to adulthood (Stier et al., 2022).

## How often is developmental plasticity adaptive?

There is sometimes a presumption that any change in phenotype in response to a changing internal state or external environment is adaptive. This is especially so in the case of transgenerational plasticity (TGP), where it is all too easy to make the assumption that, if the process is considered to be one where parents are modifying the phenotype of their offspring, it must be for beneficial reasons. This is evident in the non-neutral terminology sometimes used to describe these plastic responses, such as predictive adaptive responses (Gluckman et al., 2005; Harmon et al., 2023) in the human and biomedical literature, while in other organisms they may be referred to as anticipatory maternal (or parental) effects, where the parent is ‘anticipating’ the environment that will be faced by the offspring (Raveh et al., 2016; Uller et al., 2013). But it is also worth noting that, in the human context, a completely separate literature from the one that talks about predictive adaptive responses refers instead to maternally derived stress (MDS), which implicitly presumes that offspring will be harmed if the mother was stressed during pregnancy or early rearing (Aizer et al., 2016; Sheriff and Love, 2013).

In some cases the TGP does indeed appear to be adaptive. For instance, a perceived higher population density (whether real, or experimentally induced by acoustic playback of territorial calls) in North American red squirrels (*Tamiasciurus hudsonicus*) triggers the production of glucocorticoid hormones in breeding females; in turn, these stimulate faster offspring growth – which is adaptive in a high-density environment (Dantzer et al., 2013). In a more controlled laboratory setting, a cross-fostering experiment using the European earwig (*Forficula auricularia*) showed that offspring facing the same food level (whether high or low) as their mothers had experienced had a higher survival rate, suggesting that mothers modified the phenotype of their offspring to that suited to the current environment (Raveh et al., 2016).

However, whether developmentally plastic responses are actually adaptive will partly depend on whether it is possible to predict the future environment. In theory, any phenotypic adjustment could either be based on cues that will predict future conditions (e.g. a cold spring might indicate that food may be limiting later in the year), or on the assumption that the current and future environments will be similar (environmental ‘matching’). The match between current and future environments depends on the organism's expected lifespan relative to the temporal scale of environmental fluctuations (Bateson et al., 2004), and will generally be weaker with TGP than with within-generation plasticity (WGP) responses (Bell and Hellmann, 2019; Sheriff and Love, 2013). Recent evidence from a long-term study of experimental evolution supports the hypothesis that developmental plasticity will be less favoured if the future environment is unpredictable (Leung et al., 2020); this also supports the idea that plasticity carries costs (see next section) and so only evolves when it is likely to be beneficial.

Despite the intuitive appeal of the idea, and the positive results from some studies (e.g. Raveh et al., 2016), a meta-analysis a decade ago concluded that there is little widespread evidence that offspring do better when their environment matches, rather than mismatches, that of their parents (Uller et al., 2013). This is partly because the design of experiments to test these ideas is very problematic, as offspring reared in good conditions are likely to perform better overall (the silver spoon effect; Monaghan, 2008), masking the impact of whether parental and offspring environments are matched (Engqvist and Reinhold, 2016). A more recent assessment concluded that the evidence is still not clear whether TGP is more usually adaptive than maladaptive (Sánchez-Tójar et al., 2020); this is clearly an area in which rigorous experiments and analyses are still needed (Engqvist and Reinhold, 2016).

In the case of altered trajectories, the argument for the response being adaptive lies in the fact that there are often short-term fitness advantages to regaining the original trajectory, if the original target phenotype is still presumed to be the optimal one for the situation. Although there may have been a period of adversity during development, this has not altered the perception of what adult phenotype will have the highest fitness. Here, there is no presumption that the developing animal needs to monitor the current environment in order to predict the future – it may have an intrinsic developmental trajectory that it is attempting to match, so that it attempts to compensate for any deviation (Klingenberg, 2019). Whether this remains the optimal strategy will then depend on what compromises are needed in order to regain that ideal trajectory. In general, strategies that provide fitness gains in early life will be favoured even if they lead to more rapid senescence, owing to the forces of selection acting more strongly earlier in life (Lemaître et al., 2015).

## What constrains developmental plasticity?

Animals can show dramatic changes in their phenotype, even after development has been completed (Piersma and Drent, 2003). Notable examples of this phenotypic flexibility include physiological and morphological remodelling in response to predictable changes in energetic demands; this remodelling can be triggered by alterations to the food supply (Secor, 2008; Wikelski and Thom, 2000) or environmental temperature (Vézina et al., 2017), or the demands of a strenuous migration (Piersma, 1998). These reversible changes suggests that the basic body plan can be very flexible (Piersma and Drent, 2003). This prompts the question as to why the development of that body plan appears to be relatively constrained during development: given that plasticity has the potential to be advantageous, why don't animals show more developmental plasticity?

The answer must lie in the potential costs to phenotypic plasticity (Auld et al., 2010; DeWitt et al., 1998; Murren et al., 2015). These costs can be separated into two broad categories: those associated with retaining the capacity to be flexible (‘maintenance costs’), and those associated with putting that flexibility into action and actually generating the new phenotype (‘production costs’) (Auld et al., 2010). Maintenance costs include the expense of maintaining (regardless of the environment) the sensory and neural systems that are needed to detect and respond to the cues that trigger a response, and maintaining the machinery needed to produce more than one phenotype. Production costs include the cost of remodelling the phenotype; it may be more expensive to change the target phenotype or trajectory during the course of development than to have had that target or trajectory from the outset.

Both production and maintenance costs could apply equally to adjusted trajectories as adjusted targets, but neither form of cost is easy to measure directly. Both are presumed to include an energy cost, whether to be in readiness for, or to mount, a plastic response (Auld et al., 2010). There may be nutritional considerations other than energy: indirect evidence of this comes from the finding that diet quality (in terms of fatty acid composition) can influence the extent of physiological plasticity, through constraining the composition of cellular membranes (Hardison et al., 2023). Perhaps surprisingly, given their often hidden nature, it is easier to measure the production costs of adjusted trajectories than adjusted targets. For instance, a cost of an accelerated growth trajectory is the need for a higher food intake: this can result in more time spent in riskier foraging areas and/or less attention devoted to anti-predator vigilance, leading to a measurably higher mortality from predators (Gotthard, 2000).

Adjusted trajectories can also carry diverse long-term costs, which may become apparent only after the period of growth and development has ended and the trajectories have converged on a superficially similar adult target phenotype. In experiments that trigger compensatory growth by manipulations of food availability, it can be impossible to assign long-term costs to the accelerated growth rather than the earlier food restriction. This short-coming was avoided in a long-term study of three-spined sticklebacks in which the initial period of slowed growth was achieved by imposing unseasonably cold temperatures; at the end of this ‘cold spell’ the fish were thus just small for the time of year, rather than deprived of food (Lee et al., 2010). Moreover, by having a control group experiencing typical ambient temperatures but also a third group given unseasonably warm conditions, it was also possible to have a test of whether animals that were large for the time of year were able to benefit from being ‘ahead of schedule’. Once all fish were returned to typical ambient temperatures, the group that were small for the time of year showed accelerated growth, while those that were ahead of schedule slowed down, so that all three groups converged on the same mean size by the time of reaching sexual maturity (Lee et al., 2010). However, although the three groups were statistically indistinguishable in morphology at adulthood, the fish that had undergone accelerated growth subsequently produced smaller eggs, were slower to build nests and had a reduced period of sexual ornamentation (Lee et al., 2012), had a faster decline in swimming performance over the breeding season (Lee et al., 2010) and a shorter lifespan (Lee et al., 2013), while those that had slowed down growth relative to controls showed the opposite (beneficial) trends.

A similar phenomenon of long-term costs of accelerated growth was found when damselfly larvae were triggered to grow faster by a late-season photoperiod: they achieved a near-normal target adult size despite the compressed period of development (De Block and Stoks, 2004), but as adults had reduced immune defences and fat stores, indicating a poorer resilience (Stoks et al., 2006a,b). An analogous situation is faced by individuals that have, because of resource limitations, ‘cut corners’ in order to achieve a particular adult form. Thus, the male sticklebacks that attempt to maximise their sexual coloration at the start of the breeding season despite having a low intake of dietary carotenoids are successful in early matings, but their throat coloration soon fades and so these males are increasingly rejected by females as the breeding season progresses (Lindström et al., 2009). Similarly, the lightweight replacement claw grown by male fiddler crabs may be of normal size and so equally attractive to females (Backwell et al., 2000), but it is ineffective in fights with rival males (Lailvaux et al., 2009) and so results in poorer reproductive success because the holder cannot retain a good territory (Reaney et al., 2008). Different developmental trajectories can thus result in apparently the same adult external morphology, but lead to very different life histories.

**Fig. 2.**
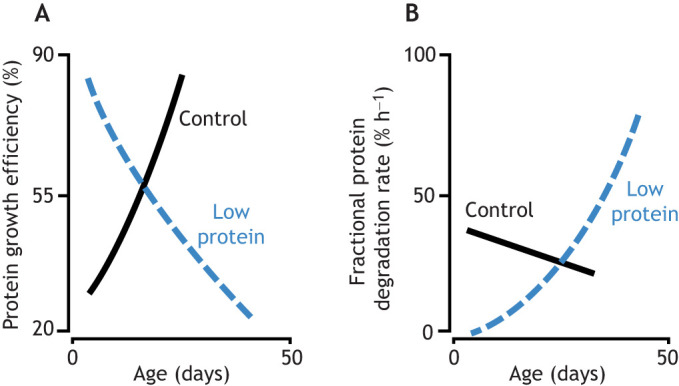
**An example of the costs of plasticity, and the trade-off between early benefits and later costs.** Echinoderm larvae on a very low protein diet (one-tenth the protein intake of controls) can initially achieve a relatively high growth rate (one-half that of controls) through (A) having a higher protein growth efficiency (% of assimilated protein that is devoted to growth). However, this high protein growth efficiency is only possible through (B) almost eliminating maintenance of existing proteins (quantified as protein degradation rate). This strategy cannot be sustained, and eventually protein maintenance costs rise steeply above those of controls, causing growth efficiency to drop sharply. Data taken from Ellison et al. (2021).

The mechanisms underlying these trends can be hard to unravel. The shorter lifespan of fast-growing animals may be due to the link between telomere dynamics and senescence. Faster growth at the embryo stage (Stier et al., 2020; Vedder et al., 2018) or in post-natal life (Monaghan and Ozanne, 2018; Salmón et al., 2021) has been shown to lead to faster telomere attrition, which can trigger cellular senescence and is associated with a reduced lifespan (Wilbourn et al., 2018). In revealing an among-species negative relationship between embryo growth rate and rate of ageing in birds and mammals, Ricklefs (2006) speculated that rapid growth could only be achieved at the expense of reduced quality control. This would result in a greater risk of ‘system failure’ later in life, explaining the accelerated ageing of species with faster early growth rates. Some indirect evidence for this comes from an intriguing study of larval growth in the echinoderm *Dendraster excentricus* (Ellison et al., 2021). Reducing the protein intake of larvae by 90% relative to that of controls caused a smaller reduction in their growth rate (by 50%) than might be expected. However, the larvae on the low protein diet were only able to achieve this growth rate by diverting virtually all of the ingested protein into growth, with almost none allocated to maintenance (unlike in controls) (Fig. 2A). This appeared to be an unsustainable pattern of resource allocation, because the relative allocation to maintenance then had to rise steeply (and above that of controls) as the larvae became older (Fig. 2B; Ellison et al., 2021). This suggests that systems may fail, triggering heavy repair investment, if there is no regular allocation to maintenance (as is known by all home owners!). Given its apparent costs, the extent of developmental plasticity may thus be traded off with other traits. This may explain why there is so much intraspecific variation in the capacity for phenotypic plasticity (Norin and Metcalfe, 2019). Burraco et al. (2022) found that the extent of morphological plasticity in spadefoot toad (*Pelobates cultripes*) tadpoles in response to predator cues varied among families, but the families with the greatest plasticity also had the highest levels of oxidative damage, suggesting that investment in plasticity came at a cost of increased oxidative stress.

A further constraint on plasticity is the speed at which plastic changes can be achieved, and whether these can match the speed of environmental change (Fox et al., 2019). If environmental conditions change rapidly, then it may not be possible for organisms to adjust their phenotype quickly enough to match the prevailing situation, making plasticity suboptimal (Einum and Burton, 2023; Nilsson-Örtman and Brönmark, 2022). There is then selection on the speed, as well as the extent, of plasticity (Burton et al., 2022). Although this argument has been developed in the context of reversible plasticity, it applies equally to developmental plasticity – with the further proviso that the natural speed of development cannot be so fast that it is impossible to accommodate a change in either developmental trajectory or developmental target. For instance, it is possible that a relaxation of adverse conditions occurs too late in development to allow time for a compensatory adjustment to the trajectory before the normal period of growth or development has finished. What is surprising (and as yet unexplained) is the fact that ‘plasticity rates’ (the speed of change of the phenotype upon encountering a new environment) vary so much among taxa: analysis of reversible plasticity in response to temperature change shows, for instance, that amphibians and reptiles are capable of changing their phenotype far faster than, say, fishes or insects (Einum and Burton, 2023). The reason for this is unknown, but may be related to the relative thermal predictability of the environment in which these animals have evolved (Einum and Burton, 2023). Whether such taxonomic differences also apply to developmental plasticity remains to be seen.

## Perspectives and future directions

I hope I have convinced the reader that it is necessary to look beyond the final phenotype when studying and quantifying developmental plasticity: the route by which that phenotype was reached is also relevant. But we still need to explore in more detail the extent and consequences of that plasticity in trajectory. For instance, it would be instructive to investigate the following. First, the controlling mechanisms underlying plasticity in developmental trajectories, and what sensory and feedback systems are involved. Second, how this plasticity varies among tissues, as well as at the whole-body level (for instance, at what points in the development of different tissues does cell division cease, so constraining the scope for plasticity?). Third, how this plasticity varies among taxa – is it greater in ectotherms than in endotherms (where growth is more independent of ambient temperature)? Is it inversely related to the predictability of environmental conditions? And fourth, what are the costs of varying the rate of development at the molecular and cellular level – is it a general finding that accelerated development leads to greater molecular damage (owing to higher levels of oxidative stress and/or less investment in cellular maintenance)? If so, can markers of molecular damage at the end of the developmental period be used as measures of the deviation in developmental trajectory?

Consideration of these and related questions should allow a more complete understanding of the selection pressures and constraints acting upon development, and hence the extent to which it is plastic.
